# Standardized Gene Nomenclature for Chicken Scavenger Receptors

**DOI:** 10.17912/micropub.biology.001559

**Published:** 2025-04-11

**Authors:** Coltton Kirkpatrick, Fiona McCarthy

**Affiliations:** 1 College of Animal Comparative and Biomedical Sciences, University of Arizona, Tucson, Arizona, United States

## Abstract

Scavenger Receptors (SCARs) are a heterogeneous group involved in innate immunity and immunomodulation. We provided standardized nomenclature for chicken SCAR genes in line with other vertebrate nomenclatures. Chicken SCAR genes orthologous to human genes are assigned the same gene names and symbols. Novel SCAR genes were assessed to determine their appropriate class and assigned nomenclature in line with this classification. Notably, chicken has expansions of the MRC and DMBT genes, and the number of these paralogs varies across Sauropsida species. In addition, we identified and classified a further 15 novel SCAR genes, predominantly SCAR class I genes.

**Figure 1. DMBT and MRC gene expansions in Sauropsida compared to human:**
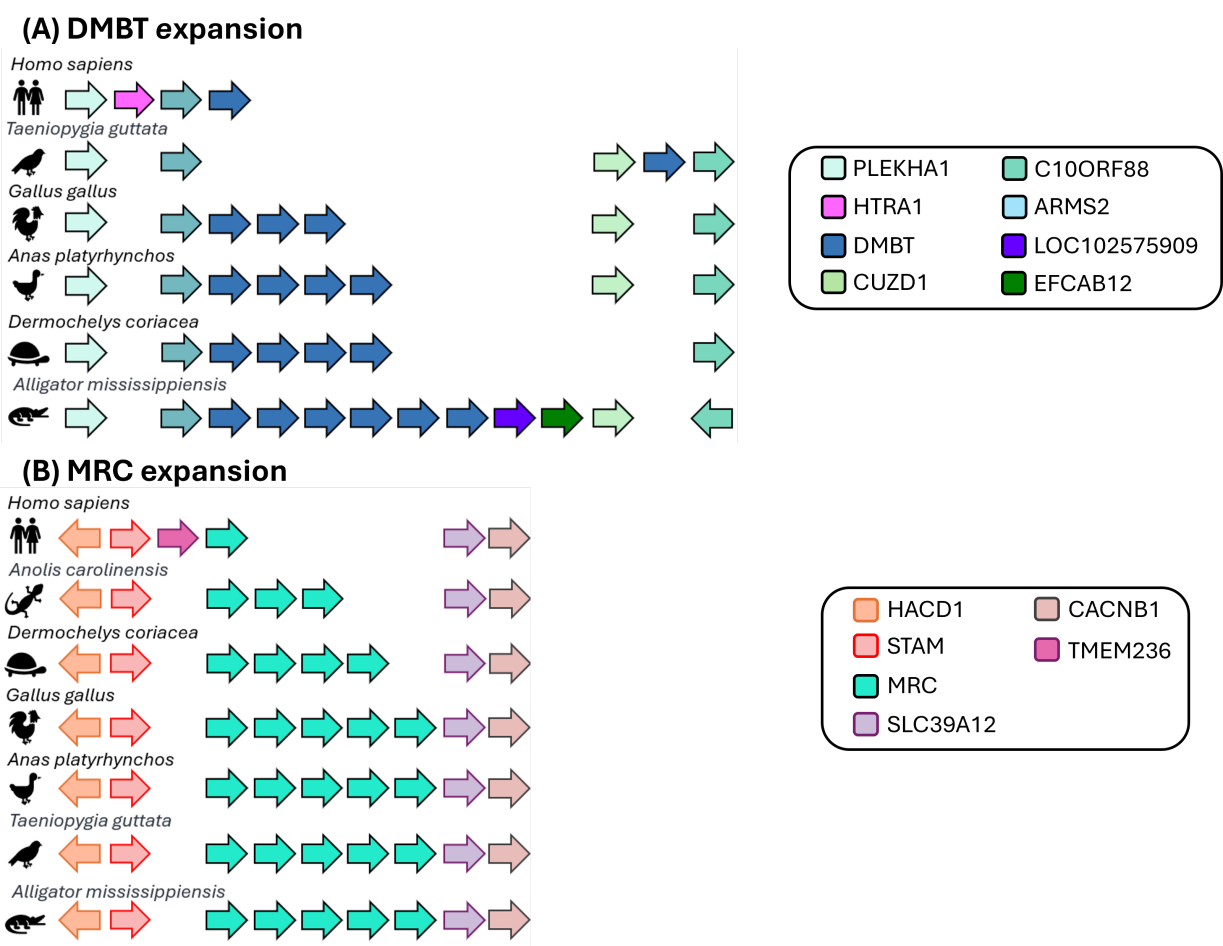
Syntenic regions of selected Sauropsida species are depicted in comparison to the orthologous region in the human genome. Protein coding genes are represented by arrows, with arrows of the same color representing paralogs of the same gene. (A) The expansion of DMBT paralogs. While humans have a single copy of DMBT, the number of DMBT paralogs varies across individual Sauropsida species. The synteny of protein coding genes on either side of the DMBT gene expansion is well conserved. (B) The expansion of MRC paralogs. This figure shows the expansion of MRC paralogs amongst Sauropsida species compared to the single copy of MRC identified in the human genome.

## Description

Scavenger receptors (SCARs) are a structurally diverse group of soluble and transmembrane surface proteins involved in immune responses, including adaptive immunity, cytokine production, and innate immunity where they function as pattern recognition receptors (Taban et al, 2022). In mammals, SCARs are divided into twelve classes of scavenger receptors (designated A-L), based on their protein structure and nucleotide sequence (Krieger, 1997; PrabhuDas et al, 2014; PrabhuDas et al, 2017). Our experimental approach is to identify SCARs in the chicken genome and assign them standardized gene nomenclature in line with SCARs from other vertebrate species (Jones et al, 2023). We identified orthologous genes to known human SCAR genes and identified novel chicken SCAR receptors based upon sequence and motif searches and associated publications about gene function. These chicken SCAR genes were assigned standard gene nomenclature. We confirmed the initial report by He et al. (2009) that only four classes of scavenger receptors are found in chicken (class A, class B, class F, and class H) and 17 of the 29 human SCAR genes have a clear chicken ortholog, which we assigned nomenclature to reflect this relationship. Additionally, we identify 22 novel chicken SCAR genes.

In Sauropsida, we identified a mannose receptor C-type (MRC) gene expansion varying in size with each species. Chickens have four additional MRC paralogs in addition to MRC1 and MRC2. We propose naming these paralogs MRC3-MRC6. Human MRC1 (Gene ID: 4360) is associated with macrophages and human MRC2 (Gene ID: 9902) is associated with fibroblasts. While there is no cellular data available for corresponding expression of chicken MRC gene products, chicken MRC1 (Gene ID: 420516) and MRC2 (Gene ID: 419950) expression is greatest in tissues with high levels of macrophages and fibroblasts, respectively. As expected, the chicken paralogs show differing patterns of tissue expression. Chicken MRC3 (Gene ID: 771888) and MRC4 (Gene ID: 420515) are both expressed predominantly in skin while chicken MRC5 (Gene ID: 771876) and MRC6 (Gene ID: 428421) are expressed in both adipose and skin. Previous work identified that MRC3 (formerly MMR1L4) is expressed on peripheral blood monocytes (Maxwell et al, 2024).

While there is a single copy of human DMBT1 deleted in malignant brain tumors, chicken has three DMBT-like genes. Motif analysis of these DMBT-like genes shows only two genes (Gene ID: 101750892 and Gene ID: 107053714) produce proteins with the same scavenger receptor CUB and ZP domains which are also found in mammalian DMBT1 genes (Ligtenberg et al, 2010). The third gene (Gene ID: 107049019) has only three scavenger receptor domains and is missing a CUB domain. Based upon this analysis, we propose that the two chicken DMBT genes with conserved motifs be renamed as DMBT2 (Gene ID: 101750892) and DMBT3 (Gene ID: 107053714) while the remaining DMBT-like gene with the missing CUB domain can provisionally be called DMBTL2 (Gene ID: 107049019). The current chicken DMBT1 (Gene ID: 426819) has no synteny with mammalian DMBT1 genes. Motif and transmembrane domain analyses show that chicken DMBT1 protein has an intracellular C-terminus domain and an extracellular region containing 12 scavenger receptor domains. This structure matches the scavenger receptor class I proteins. Noting that the symbols SCARI5-24 are reserved for novel bovine genes (Prabhudas et al, 2014) and we propose this gene be renamed SCARI25 scavenger receptor class I member 25. Four additional chicken genes which are currently called ‘deleted in malignant brain tumors 1 protein-like’ should also be reclassified as SCAR class I (Gene IDs: 121106432, 426820, 121113258, 107049129). Likewise, two additional genes currently called soluble scavenger receptor cysteine-rich domain-containing protein SSC5D-like (Gene ID: 101748207 and Gene ID: 101747860) should also be reclassified as SCAR class I. Analysis indicates that three chicken class I genes lack a transmembrane domain and are secreted. Notably, the human DMBT1 protein (which is classified as SCAR class I) also exists in a secreted form (Mollenhaur et al, 1997). We note that Gene ID: 107053766 has a scavenger receptor cysteine-rich (SRCR) domain, making it a member of the closely related SRCR gene group. An additional gene (Gene ID: 121113282) appears to be incorrectly annotated and may be better represented by ENSGALG00010026037 or ENSGALG00010026019 genes; assigning corrected nomenclature may need to await improved annotation in this region.

We identified six “antigen WC1.1-like” genes on the reference chicken genome. While not found in humans, other mammals (notably ungulates) contain SCAR WC1 genes. Analysis of gene synteny blocks and gene order between chicken ( *Gallus gallus* ) and *Bos taurus* identified no synteny. Five of the chicken WC1-like genes are predicted to be SCAR type I, and we propose nomenclature to reflect this classification. The other gene (Gene ID: 101750114) has a scavenger receptor cysteine-rich (SRCR) domain and is not included in further analysis. We note an additional seven WC1-like genes identified from the closely related leghorn genome which are likely to represent additional SCARI genes. However, due to the relatively poor annotation in this region of the leghorn genome, these genes are not included in this study.

Current gene nomenclature indicates that chicken CD163 (Gene ID: 418303) and CD163L (Gene ID: 426826) are not orthologous to human CD163 (Gene ID: 9332) and CD163L (Gene ID: 283316), and motif and protein structural analyses support this. Instead, these genes are most closely related to SCAR class I receptors and we propose chicken CD163 be renamed SCARI37 scavenger receptor class I member 37 and chicken CD163L be renamed SCARI38 scavenger receptor class I member 38.

Chicken PIT54 (Gene ID: 395364) was first identified in a study identifying acute inflammatory responses in chicken (Iwasaki et al, 2001). Protein sequencing identified PIT54 as a member of the scavenger receptor cysteine-rich (SRCR) group, related to SCAR proteins. Based upon our analyses, chicken PIT54 is a SCAR class I gene. Therefore, we propose chicken PIT54 be renamed SCARI39 scavenger receptor class I member 39.

Overall, chicken SCARs are represented by a different set of genes between humans and chickens. While humans and chickens have 17 conserved orthologs, chickens are missing an additional 10 SCAR genes found in humans but have 22 novel SCAR genes not found in humans. Moreover, the variation in the number of MRC1 and DMBT gene copies amongst Sauropsida indicates that the diversity of this gene family extends to other species in this taxonomic class. Careful manual review of the SCAR genes will be required across multiple species to ensure that gene symbols and names are clearly and consistently assigned to support comparative studies of these key immune receptors. The genes analyzed in this study and the proposed nomenclature have been submitted to the Chicken Gene Nomenclature Committee (CGNC) for review.

## Methods

Human SCAR genes are identified from the HUGO Gene Nomenclature Committee (HGNC) gene group (Seal et al, 2023). These human SCAR genes serve as the base sample set for identifying chicken SCAR genes. Chicken orthologs are identified using the National Center for Biotechnology Information (NCBI) ortholog database (Goldfarb et al, 2025). If a chicken ortholog is found, the gene synteny is manually checked and confirmed, and then the human SCAR gene nomenclature is transferred to the chicken gene. Chicken gene synonyms are manually identified by reviewing literature associated with the chicken gene. In instances where no chicken orthologs were identified, the NCBI orthologs were reviewed to determine if the human SCAR gene is found in other bird species.

To identify chicken SCAR genes with no human ortholog, we did BLASTp searches against the chicken subset of the NCBI non-redundant protein database (NRPD) using human SCAR protein sequences (Camacho et al, 2023). To confirm the matches were SCAR genes, we did motif analysis using the conserved domain database search at NCBI (Wang et al, 2023) and SCAR subclasses were confirmed based upon this and transmembrane predictions from DeepTMHMM 1.0 (Hallgren et al, 2022). To determine the relationships of these proteins with the other chicken SCAR proteins, we did phylogenetic analysis. To create a phylogenetic tree, amino acid sequences were aligned using Geneious Alignment, and this alignment was used to create a phylogenetic tree using Jukes-Cantor, bootstrapped 100 times (Geneious Software version 2024.0.7). The results of the phylogenetic analysis were combined with manual review of gene synteny and protein structure analyses as well as tissue and single cell type expressions. Expression of chicken gene products was determined by using FAANGMine (faangmine.elsiklab.missouri.edu) to search for combined tissue RNA-seq datasets, while tissue and cell expression data of human genes was accessed from the Human Protein Atlas (Uhlén et al, 2015). Lastly, a review of available literature for these genes is conducted to determine any aliases or additional names used in the associated literature.
